# Phase, microstructure and service character of as-deposited and short-time heat-treated Ni-Mo alloys with mixed state

**DOI:** 10.1371/journal.pone.0249875

**Published:** 2021-05-21

**Authors:** Hong Xu, Ning Li, Qinghua Zhuo, Lirong Lu, Weizeng Chen

**Affiliations:** 1 Xingzhi College, Zhejiang Normal University, Jinhua, China; 2 College of Engineering of Zhejiang Normal University, Jinhua, China; College of Engineering, University of Saskatchewan, CANADA

## Abstract

Considering the amorphous and nano-crystalline cluster structure and their activity, on the basis of the mixed structure Ni-Mo alloys, the crystallization kinetics of the alloys and the performance of the alloys after heat treatment with different mixed structure were studied. The phase structure and composition were determined by X-ray powder diffraction. The crystallization activation energy of the mixed structure was obtained by differential scanning calorimetry. The electrochemical activity of the mixed structure alloy was determined by electrochemical analysis. The experimental results show that the structural stability of the mixed-structure alloy is better, but the crystallization activation energy is much lower than that of the amorphous alloy. The crystallization process consists of a meta-stable structure transition and a new phase formation. The electrochemical properties of the alloy indicated that the alloy with the mixed structure has higher electrochemical activity, with higher hardness and better corrosion resistance, which results from the large true contact surface and the large number of active centers in this material structure.

## 1. Introduction

Amorphous alloys have been widely confirmed in the field of new materials due to their excellent physical and chemical properties with the characteristics of long-range disorder, short-range order and metastable structure. The alloys with high corrosion resistance, excellent electrochemical activity and lower hydrogen evolution over-potential etc., have been applied and developed in the aerospace industry, electromagnetics, new energy and chemical industries [1–4]. The long-range ordered and short-range ordered structure of nanocrystal materials is different from the conventional ordered and amorphous disordered structure. This owns the characteristics of amorphous state and quantum size, which leads to the unique and excellent properties, such as high corrosion resistance, high catalytic activity and high toughness [5–8]. Combining the characteristics of amorphous and nanocrystal materials, the amorphous and nanocrystal composite coating was obtained by one-time controllable parameters through the electrodeposition technology, composed with the characteristics of amorphous structure and the nanocrystals. Among the reported electrodeposited alloy materials, Ni-Mo alloys are the excellent comprehensive material with outstanding physicochemical characteristics [9, 10]. The research on amorphous and nanocrystal Ni-Mo alloys indicates that the reactivity and stability of Ni-Mo alloys for electrocatalytic hydrogen evolution are closely related to the existence state of phase composition and microstructure. As hydrogen evolution cathodes, it shows an excellent electrochemical property due to the inner amorphous structure of the material [11–15]. The microstructure in the alloy determines the macroscopic properties of the electrode. The main feature of the amorphous state is metastable state, which directly determines the service life of the electrode in practical applications. The crystallization behavior of the amorphous state will determine the structural phase of the material, affecting the critical materials own structure and performances. For the alloy with amorphous and nanocrystal mixed structure, the existence of nanocrystals will inevitably affect the crystallization process of the amorphous alloy, the internal phase composition, amorphous content and specific surface area of the material [16], which will determine the physicochemical properties of the electrode.

In this paper, a certain amount of stabilizer and modifier are added to the base deposition solution of the amorphous Ni-Mo alloy coating to improve the deposition performance of the deposition solution. The co-coating composed with amorphous-nanocrystal mixed structure is obtained one time. Thermal analysis of the mixed structure coating was carried out by differential scanning calorimeter to obtain the crystallization transition temperature of the mixed structure. Different heat treatment temperatures effects on micro-hardness, corrosion resistance and electrical-chemical properties of the coating were investigated. The relationship between the amorphous content, composition, specific surface area and the electrode activity of the co-coatings were established and discussed.

## 2. Experimentals

### 2.1 Experimental materials and preparation

Without consideration of the influence of other impurity elements, the electrolyte was prepared with double distilled water and all the analytical reagents were of analytical grade (Sinopharm Chemical Reagent Beijing Co., Ltd., Beijing, China). The main component content was given separately as 48.0 g·L^−1^ NiCO_3_·2Ni(OH)_2_·4H_2_O, 12.0 g·L^−1^ Na_2_MoO_4_·2H_2_O, 50g·L^−1^ Na_3_C_6_H_5_O_7_·H_2_O, 18g·L^−1^ H_3_BO_3_, 5g·L^−1^ NH_4_Cl, 2.4g·L^−1^ Saccharin, 24.0 g·L^−1^ additives (6.0 g·L^−1^ ascorbic acid, 4.0 g·L^−1^ sodium dodecyl benzene sulfonate, 10.0 g·L^−1^ and ethylenetetraaminetetraacetic acid). The additives were prepared ourselves. Deposition conditions: pH is 10.5 ± 0.2, adjusted with 5% ammonium citrate solution. Current density was 5.6± 0.1 A·dm^−2^, WYK-305 co-current voltage-stabilized source (Handan Dawei Electroplating Equipment Co. LTD., Handan, China) was used to control the working current density. Temperature is 33 ± 2°C, bath temperature was controlled by DF-101S collector magnetic heating stirrer (Handan Dawei Electroplating Equipment Co. LTD., Handan, China). The deposition time was 30 min.

During the deposition process, pure nickel plate was used as anode (*w*_Ni_ = 99.99%) and copper plate as the cathode. Before electrodeposition, the oxide film on the surface of the sample was removed. Epoxy resin was sealed on the back of the cathode. Then, the samples were degreased and activated with acetone and 10% dilute hydrochloric acid and 10% dilute nitric acid mixed solution and rinsed with distilled water. The surface area ratio between cathode and anode was 1:4, separated by 2.5–3.0 cm distance apart, submerged at the depth of 5–6 cm parallel to each other.

### 2.2 Composition test

The deposited coating materials were peeled off from the substrate and grinded to powder. The phase components and structure of the coating were measured by SMART APEX II single crystal X-ray diffractometer (Bruker SMART APEX II, BrukerSmart, Rheinstetten, Germany). Among them, Cu Kα is used, the tube voltage is 35KV, and the scanning speed is 4°/min. The grain size is calculated using the Scherrer Equation [17]. The composition of the formed alloy is set to 100, and the coating is linearly fitted to the crystal. The proportion is determined according to the area of peak strength of crystalline phase, and the crystallinity of the formed alloy can be estimated. Its calculation formula is as follows:

$$\delta =k\frac{I_{A}}{I_{A}+I_{B}}\times 100\%$$ (1)

Where I_A_ is a crystalline phase, I_B_ is an amorphous phase, and K is a correction factor. Regardless of the actual influencing factors, k = 1 is generally considered.

Component analysis of the coating was carried out by complexometric titration. Ni^2+^ was titrated with 0.008mol/L EDTA standard solution by electroplating low carbon steel as cathode, stripping the obtained coating, dissolving in dilute nitric acid, adjusting pH to 8–9 with ammonia water, and using ammonium urea as indicator.

### 2.3 Thermal analysis experimental method

The synchronous thermal analyzer STA 449C DIL402C Luxx (NETZSCH, Germany) was used for thermal analysis and heat treatment. The heating rates were 5, 10, 20 and 30°C/min, respectively. Ar_2_ atmosphere was used to protect the heating process, and the flow rate was 20 ml/min. After rising to the required temperature, the temperature was kept constant for 30min, and then cooled naturally in Ar_2_ atmosphere. According to the relationship between peak temperature Tp and heating rate of DSC, the activation energy E of crystallization was calculated by the formula as follows.

$$d\left[\ln\left(\frac{\phi }{T_{p}^{2}}\right)\right]/d\left(\frac{1}{T_{p}}\right)=-\frac{E}{R}$$ (2)

### 2.4 Determination of microhardness of coatings

The hardness of the coatings was tested by Shanghai 71–1 microhardness tester. The microhardness tester measures the diagonal length of the indentation remaining after the diagonal indentation of the diamond drill angle cone indenter is pressed into the measured object under 1-1000gf load by optical amplification. The formula for calculating hardness is as follows:

HV=2Psin(α2)/d2=1854.4P/d2 (3)

In the formula, HV-Vickers hardness value (kgf/mm^2^), the angle between two opposite planes of A-Square quadrangular pyramid (specified as 136°); P is load (gf); d is indentation diagonal length (μm). The simple size is 60×10×10(mm), coating thickness about 20–30μm.

### 2.5 Measurement of corrosion resistance of coatings by impregnation weight method

The so-called gravimetric method is a method to determine the corrosion rate of metal material by comparing the weight changes of the material before and after corrosion under certain conditions (pressure, temperature, medium concentration, etc.) after a certain corrosion time of the corrosive medium. For uniform corrosion, according to the condition that the corrosion products are easily removed or firmly adhered to the surface of the sample, the weight loss or weight increase of metal corrosion per unit time and per unit area can be used to express the corrosion rate respectively.

K=(W0−W)(s×t) (4)

Where K is corrosion rate g/m^2^·h (weight-increasing corrosion product not removed when K is negative, the error range of the experimental data is about 0.01mg), s sample area m^2^, t test time h, W_0_ weight (g) before test and W is weight (g) after test. The balance used for the experimental weighing was an *AE240* electronic analytical balance.

### 2.6 Electrochemical properties of alloy electrodes

A three-electrode system was used with a saturated calomel electrode as the reference electrode and a platinum electrode as the counter electrode. The copper plate substrate was used for the working electrode for the deposition-reduction process. The reference working electrode is a HMDE (Hanging Mercury Drop Electrode, SHY-217, Wuhan Gaoss union Technology Co. LTD., Wuhan, China), as it was used in the paper [10]. The working electrode was the deposited Ni-Mo composite electrode with an area of 1 cm^2^ (the electrode back was sealed with epoxy resin) to test the electrochemical properties of the obtained composites. The electrolyte concentration was 7 mol·L^−1^ NaOH solution at a temperature of 33 ± 2°C. The electrode reaction parameters were measured to determine the reversibility of the electrode reaction with the scanning speed of 5 mV/s. French Bio-logic electrochemical workstation (MTZ-35, EC-lab Ltd., Berkshire, French) was utilized while using the matching Electrochemical Workstation software system (EC-Lab) to analyze the data.

## 3. Craystalline kinetics and microstructure of deposits

### 3.1 Crystallization kinetics of coatings

**Fig 1:**
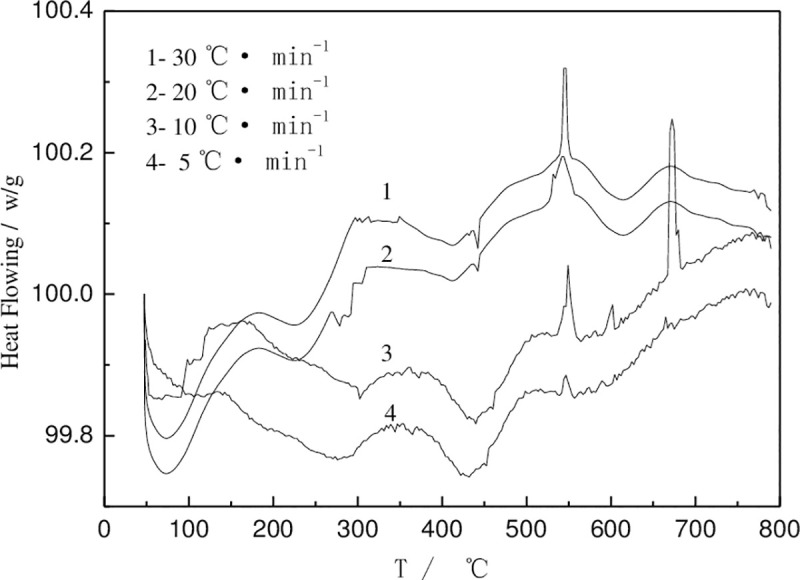
DSC patterns of the deposits Ni-Mo alloy at different heating rates.

Fig 1 shows DSC patterns of the deposits Ni-Mo alloy at different heating rates. The matching number of Ni and Mo atoms and the difference of atomic size make the system a more compact stacking structure [18–20], inhibit a long-range diffusion, increase the viscosity sharply, and inhibit the nucleation and growth of crystalline phase.

In the experiment, the reaction rate *dH/dt* and reaction rate *α* at different temperatures were measured at 8 points in the temperature range of exothermic peak (*T*_*0*_ is the starting crystallization temperature). The peak height of *dH/dt* at temperature *T* was measured, and the ratio of partial area0020030*ΔHt* of DSC curve to total area *ΔH* under DSC curve was measured. Then *dα/dT* is calculated from *dα/dT* = (*1/ΔH*)·(*1/Φ*)·(*dH/dt*). The *f(α)* and *g(α)* are kinetic functions, which are taken from references [7, 13, 14]. The activation energy *E* of crystallization can be calculated to be 3.84×10^5^*KJ/mol* by substituting the measured data into the equation for data processing.

### 3.2 Structure and phase of coatings at different heat treatment temperatures

**Fig 2:**
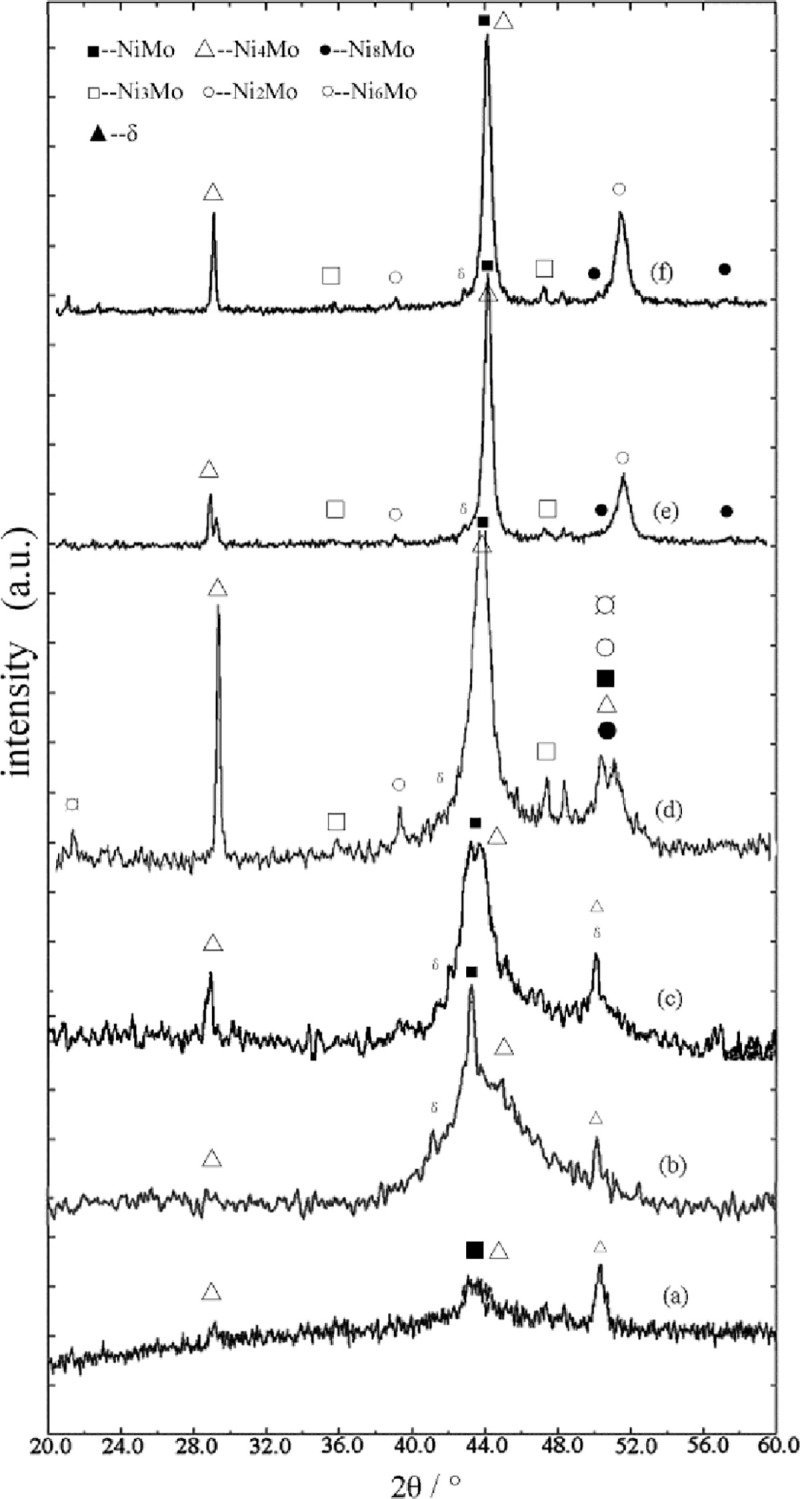
XRD curves of the deposited at different temperature (t = 30min). (a) as-deposited (b)250°C (c)350°C (d)450°C (e)550°C (f)650°C.

Fig 2 is the X-ray diffraction patterns of Ni-Mo_18.68_ alloy coating heat-treated at different temperatures for 30 minutes. Fig 2(A) is the diffraction pattern of as-deposited coating. There are pre-peaks (After the introduction of Mo element, the self-diffraction peaks caused by the correlation between Ni-Ni atoms are observed.) in the small angle part (2θ≈29.32°). The existence of pre-peaks corresponds to the orientation of crystalline growth. At the same time, the stability of the corresponding short-range chemical structure is related to the stability of amorphous alloys [21–28]. Meanwhile, there is a weaker crystallization peak (2θ≈50.38°) in the diffraction peak, which indicates that there is a crystallization phase in the deposited. However, there is only one sharpened diffraction peak. This means that there is A small amount of crystalline phases distributed on the amorphous matrix. Considering of the pre-peak and the obviously broaden diffraction peaks, the obtained alloy has the ability to form a complete amorphous [24, 25].

It can also be seen from the Fig 1 that with the increase of heat treatment temperature, the coating structure gradually changes from amorphous to crystalline phase, accompanied by the formation of new phase. Fig 2(B) shows the X-ray diffraction pattern of the coating after heat treated at 250°C. After deducting the diffraction peaks of copper on the back, the amorphous diffused peaks still exist, and a strong diffraction peak appears. Linear fitting of the crystalline state of the coatings shows that the amorphous component content about 87.34%. Therefore, the coating is a mixed structure with amorphous as the main phase [26]. Compared with as-deposited structure (Fig 2A), the content of amorphous components does not decrease, but increases. The content of nanocrystals is also counted and not been calculated separately. Meanwhile the nanocrystallines in as-deposited state still have higher activation energy. During the heat treatment process, the growth direction of endothermic metals may be reversed and the azimuth angle of atomic clusters may change, with the characteristic of a widened amorphous diffraction peak at the bottom. Thermal analysis pattern shows that the structure of the coating is relaxed at about 330°C, no new phase will appear before the crystallization temperature. The strong diffraction peak may due to the increase of the ratio of metastable phase. Fig 2(C) shows the XRD pattern of heat-treated coating at 350°C. There are pre-peaks at 2θ≈29.24° and widened and symmetrical double-peak structure diffraction peaks at 2θ≈43.12°, different from strong crystalline structure diffraction peaks and diffused amorphous diffraction peaks of "steamed" shape. The crystalline grains at 2θ≈43.12° calculated by Scherrer Equation were about 7.38 nm. And the crystallinity fitted by the crystalline state was about 11.48%. That’s mean the coatings after heat treatment are still in the mixed amorphous and nanocrystalline structure.

The diffraction intensity of the diffraction peaks of the coating heat treated at 450°C increases obviously (Fig 2D). The passive broadening at 2θ≈41~48° diffraction peaks accompanies by sharp crystalline diffraction peaks. The "steamed" shape characteristics disappear basically, and the double shoulder diffraction peaks appear at near 2θ≈51°. It is inferred that the coating with mixed structure was partially crystallized at this temperature. But many amorphous phases have not translated into crystal in a short time. The structure has not been fully crystallized and still exists. Thermal analysis patterns show that the coatings begin to crystallize at 440°C, the atomic position will rearrange and new phases will appear. At the same time, the nanocrystallines begin to grow, the crystal defects in the alloy begin to increase, and the peak position becomes obvious. The amorphous phase content of the coating is estimated to be about 37.82% after linear fitting treatment, which still occupies a part of the coating. And the coating still retains the characteristics of amorphous structure. The adsorption energy of the intermetallic compounds formed with Ni and Mo atoms is different when heated, and the thermal expansion rate is different. The crystal capacity of muddy and bonding force between metal atoms are different. When the alloy is heated, the adsorption force between them is affected by the action force of the nucleus and radius of atom and the electron cloud of the high-speed rotation of the electron outside the nucleus. The metal space structure compounds with different coordination number are formed between the two atoms. The formation and growth of these compounds require higher temperature to cause the physical and chemical transformation between the alloys. The transformation of amorphous phase is delayed by the formation and growth of new phase.

Fig 2E and 2F show the diffraction patterns of the alloys at 550 and 650°C. It can be seen from the graphs that the X-ray diffraction peaks of the coatings show obvious crystallographic characteristics with the increase of heat treatment temperature. Phase analysis of as-deposited and heat-treated coatings was carried out in Table 1, in which the metastable phase is expressed by δ.

**Table 1:** The phase composition of the deposits after heat treatment.

Composition T (°C)	NiMo	Ni_3_Mo	Ni_4_Mo	Ni_8_Mo	Ni_2_Mo	Ni_6_Mo	*δ*	structure
as-deposited	√		√				√	amp-nano
250	√		√				√	amp-nano
350	√		√				√	amp-nano
450	√	√	√	√	√	√	√	amp-nano
550	√	√	√	√	√			cry
650	√	√	√	√	√			cry
2θ / °	39.6	36.2 46.2	29.2					
	42.2		44.2					
	46.2		46.1					
Ref.	[20]	[20]	[20]	[27]	[28]	[21]	[29]	

Note: √ means existence, amp-amorphous, nano-nanocrystal, cry-crystal.

It can be seen from Table 1 that the alloy phase with mixed crystal structure obtained under deposition condition contains NiMo and Ni_4_Mo phases [19]. Heat treated at 250 and 350°C in a short time, the structure of the coating remains basically unchanged, and the metastable phase remains in the alloy structure. According to the first principle calculation in document [29, 30], it is indicated that the metastable phase maybe Ni_24_[Ni_4x_Mo_4(5-x)_]Mo_12_(0≤x≤5)with a certain stability. New phases were formed in the treated alloy after heat at 450°C, and metastable phases in the alloy structure began to change. At higher temperatures, the outermost electrons of metal atoms intensified, and the number of shared electrons changed, resulting in the formation of Ni-Mo intermetallic compounds with different spatial structures and atomic ratios. When the temperature reaches 550°C, the metastable phase in the alloy has disappeared and transformed into stable phase Ni_8_Mo and Ni_2_Mo with Ni-Mo structure. Ni_8_Mo has a body-centered tetragonal structure, similar to the spatial structure of Ni_8_Nb, and a good storage activity structure. Ni_2_Mo has Pt_2_Mo structure and shows a certain structural stability at low temperature.

Generally, the atoms in metal alloys follow a regular and orderly arrangement while growing. From Fig 2, acromion present in the diffused peak as "steamed bun" diffraction peaks of X-ray intensity curves of as-deposited amorphous alloys. The acromion indicates the chemical short-range ordered structure in amorphous structure. During heat treatment, the disappearance of the pre-peak and acromion indicates the short-range ordered growth in the alloy. The enhancement of the crystalline diffraction peak indicates that the growth process of atoms in the alloy is obvious. The internal structural atoms of metals are arranged in accordance with the spatial lattice of its own components, which improves the thermal stability of the alloy structure. The acromion characteristics of amorphous alloys remained in the coatings treated at 450°C for 30 minutes (Fig 2(D)). This may be caused by the relatively small mobility of atoms at this temperature and in a short time, and the mobility of atoms is controlled to a certain extent. Therefore, in a short time, the alloys heat treated near the crystallization transition temperature still retain the arrangement characteristics of as-deposited metal atoms. With the further increase of temperature, the temperature difference of amorphous transition crystalline state is compensated by high temperature, so the velocity of atom movement increases in a short time, the efficiency of position rearrangement increases, and the possibility of new compounds formation increases. It can be seen from Table 1 that the intermetallic compounds Ni_2_Mo and Ni_6_Mo at higher temperatures enrich the spatial structure of Ni-Mo alloys.

### 3.3 Surface morphology of alloy coating after heat treatment

**Fig 3:**
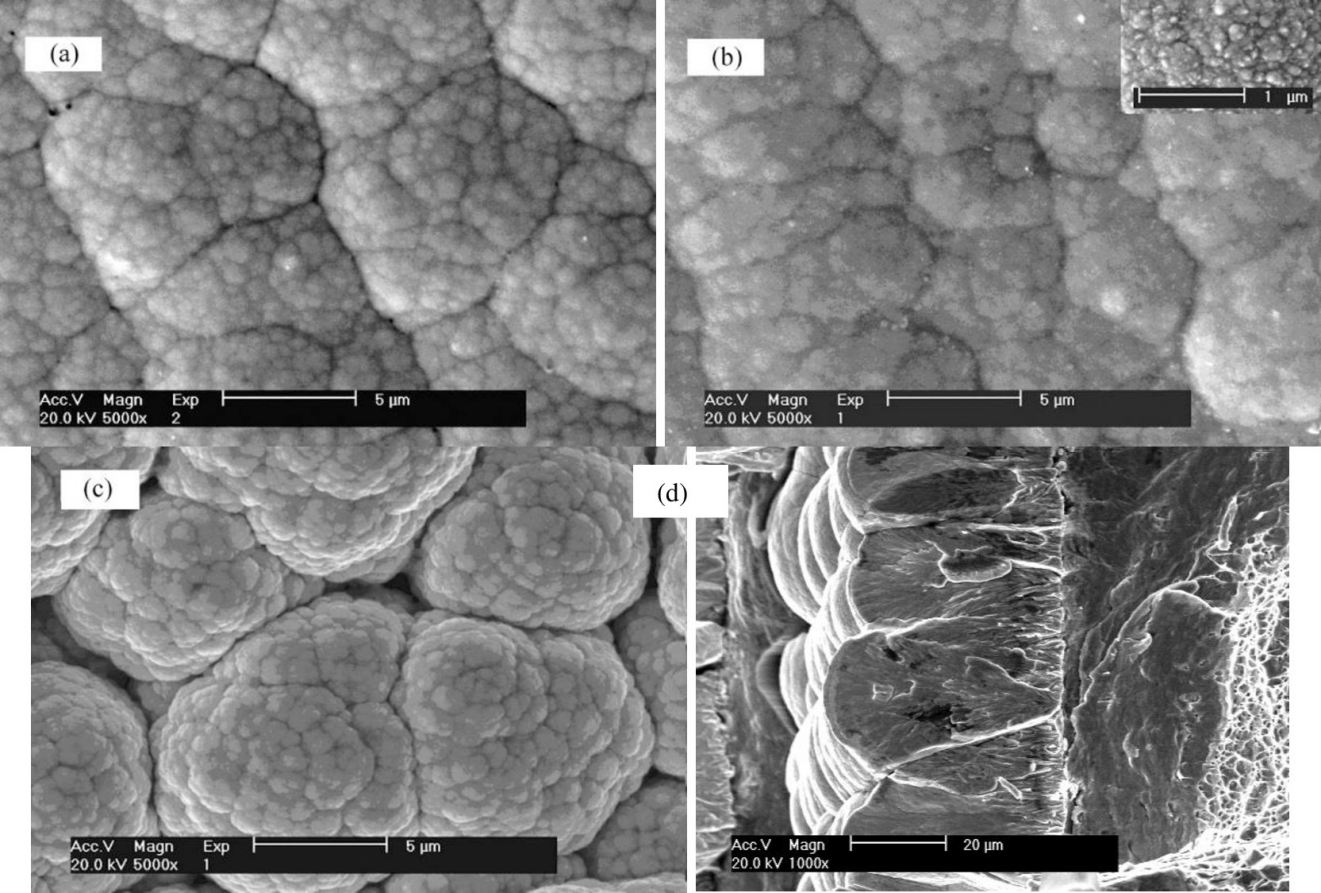
SEM images of the as-deposited and the deposits after heat treatment. (a) as deposited (b) 450°C (c) 650°C (d) cross-sectional of as-deposited.

Fig 3 shows the surface topographies of the coating after heat treatment at different temperatures. The surface of the coating is basically cellular structure. With the increase of heat treatment temperature, the boundaries of cellular particles become blurred first and then clear, and the cellular particles become smaller first and then larger gradually. When the particles grow up, the intercellular space increases and the surface of the coating becomes rough. After annealing at 450°C, the morphology of the coating is basically the same as that of the as-deposited, smooth, bright, clean and flat. The boundaries are still clear, but the brightness becomes dark. It is found that the cells are packed tightly with clear boundaries, and individual shadows appear in Fig 3(B). At this time, the diffusion and migration of atoms in the plating layer may be further accelerated due to the increase of temperature. Unstable atoms are spontaneously adjusted and rearranged under higher energy, and no longer maintain the brightness in appearance. At low magnification, the blackness of cellular particles in the surface layer increases, some boundaries become blurred. Enlarged observation shows that the cellular particles are uniformly distributed and present disintegrated small "honeycombs", which is due to the higher energy further enlarging its range of motion and the migration and diffusion movement exceeded the boundary. Therefore, after heat treatment at 450°C, the morphology of the coating is dispersed on the cotton-like as of the crystallization. With the further increase of the treated temperature, XRD diffraction patterns of the co-coating shows that the crystallization is in a large amount with more obvious intergranular boundary and the intergranular bonding is weakened.

## 4. Effect of heat treatment on the performance of mixed coatings

### 4.1 Effect of heat treatment on microhardness of coating

Table 2 shows the average grain size and Vickers hardness of the deposited and heat-treated coatings. The data show that the grain size range is fewer changes and the hardness is relatively high below 350°C. As is known to all, the characteristic of crystal grain growth of amorphous structure coating needs higher energy after structural relaxation stage. And nanocrystals themselves are unstable structures and tend to grow up when the outside world gives them higher energy. Below 350°C, the amorphous "structural relaxation" transition occurs in the coating, and the internal short-range ordered atoms will undergo structural relaxation, and the long-range atoms will diffuse. The structure will move from a metastable state to another metastable state. When the annealing temperature increases above 400°C, the grain growth trend gradually increases. After annealing at 650°C for 2 hours, the average grain size of the coating increased from 3.6nm to 45nm. The active H atoms adsorbed inside the coating combines due to endotherm and escapes from the inside of the plating layer, so that the metal atoms inside the plating layer are compactly combined, reducing the compressive stress and the relaxation force, thereby causing the microhardness change of the plating layer. But the microstructure of the coating did not change significantly. The microhardness of the coating changes greatly with the growth of crystal grains.

**Table 2:** The average grain size and Vickers hardness of the deposited and heat-treated samples.

Temperature (°C)	25	250	350	450	550	650
Average grain size (nm±0.1)	3.6±6	3.8±14	6.3±4.8	12.4±19	25.4±66	45±59
Vickers hardness (HV±1%)	856±23	740±21	846±13	883±13	725±17	542±19

The factors that generally affect the hardness of the coating are the grain structure and particle size, dislocation density and dislocations caused by impurities. The smaller the grain size of the coating, the higher the hardness, and the highest hardness of the coating at 450°C. Combined with DSC patterns and XRD patterns of the co-coatings, the formation of a high hardness Ni_3_Mo phase with the quality of a brittle, high hardness, increases the microhardness of the deposition. The coatings studied in this paper have the highest hardness at 450°C, and may also be related to the formation of metastable Ni_24_Mo_12_ phase at 350°C [22, 23]. At the same time, a new phase is formed in the coating, and the growth of the crystal grains causes a large change in the microhardness of the coating. After the crystal phase transformation of the plating, the lattice distortion is caused, the plastic deformation resistance of the plating is increased, and the hardness value is higher than that of the deposited. The recombination and position rearrangement of the internal atoms of the coating make Ni and Mo combine with the new phase Ni_2_Mo and Ni_6_Mo, and the two phases belong to the plastic phase with a certain extension. The performance, at this time, the microhardness of the coating is significantly reduced because the formation of the crystal structure forms a large number of grain boundaries and internal defects, and the growth of the nanocrystallines causes a large angle grain boundary between them, under the action of external force. The structural defects inside the coating move rapidly. The plastic phase also slows down the external force of the crystal, and the microhardness decreases.

### 4.2 Effect of heat treatment on corrosion resistance of coating

**Fig 4:**
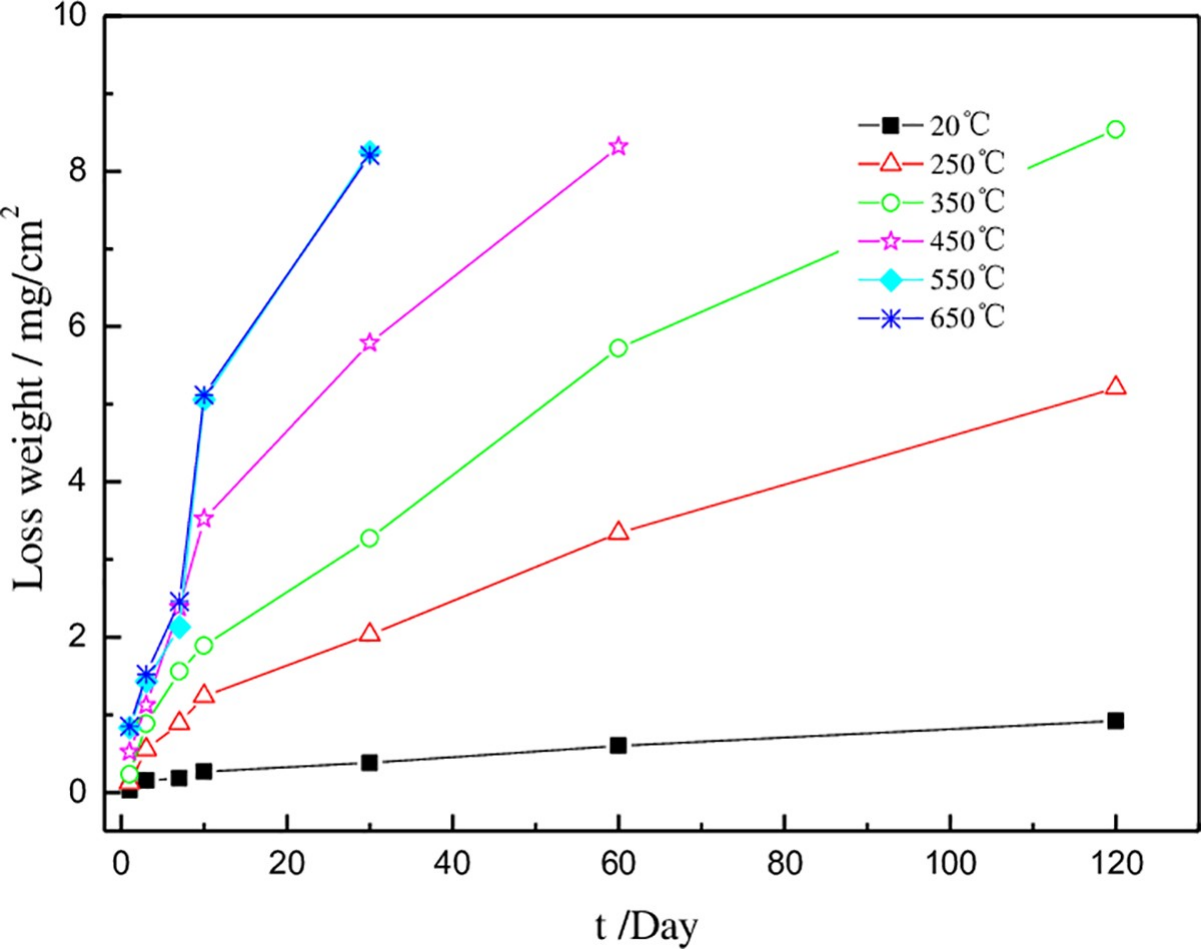
Average weight loss of the coatings in 0.4mol/L NaCl.

Fig 4 shows the relationship between the corrosion resistance of the coating alloy in 33±2°C, 0.4 mol/L NaCl solution. It can be seen from the figure that the average loss of the coating gradually increases with time until the coating is completely dissolved. The coated has less corrosion amount at room temperature, after 30 days soaked, the pattern tends to be stable, the amount of corrosion varies basically no longer changed. The corrosion loss of the coating after heat treatment at 250°C is much larger than that of the deposited. It is considered that the structural relaxation phenomenon of the coating under higher temperatures condition makes the grain boundary of the crystalline obvious. With the progress of structural relaxation, a certain new phase will be formed. These new phases reduce the corrosion resistance of the material. The corrosion resistance of the coating treated at 350°C is significantly deteriorated, and the structure of the coating becomes slack under this temperature. Some metastable phases and new nanocrystalline formed inside. The grain boundary defects reduce the corrosion performance of the material, and increase the corrosion loss amount. But the amorphous structure in the coating still has good corrosion resistance [30, 31]. When the heat treatment temperature exceeds the crystallization temperature (over 450°C), the internal defects of the nanocrystals were transferred and increased, the number of grain boundaries increased, and these structural features reduced the corrosion resistance of the coating. At the same time, the new phase produced by the recombination of Ni and Mo atoms improves the compactness of the coating, but the corrosion resistance of the coating is lowered due to the generation of many grain boundaries.

### 4.3 Effect of heat treatment on electrochemical properties of coatings

**Fig 5:**
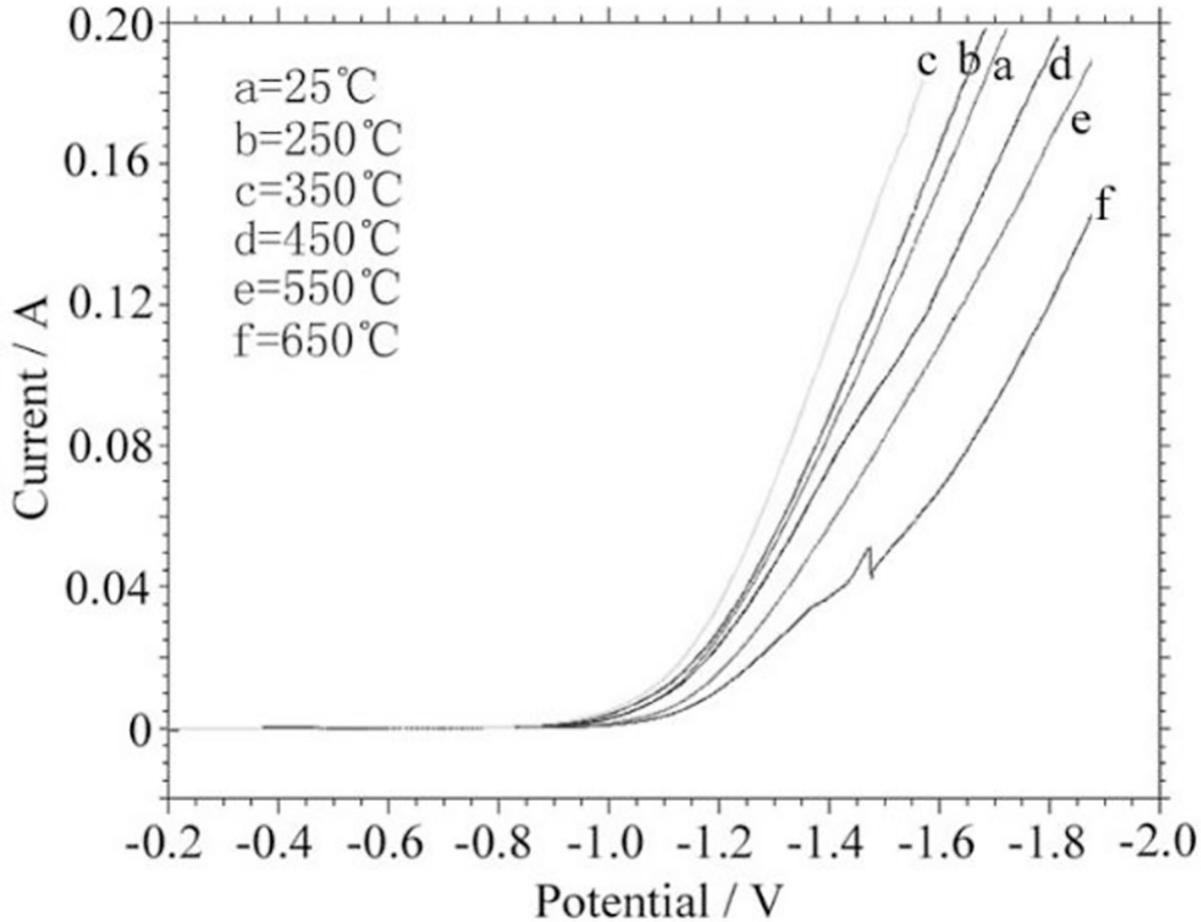
Steady electrode polarization patterns of the deposited after heat treatment.

Taken the depositons as an electrode, a steady-state polarization pattern was performed at 33 ± 2°C, 7 mol/L NaOH solution, as shown in Fig 5. The patterns show that with the heat treatment temperature increases, the chemical catalytic activity of the coatings increase first and then decrease. It is considered that the amorphous content is relatively high before the crystallization starts, and the content of short-range ordered atomic clusters in the alloy increases [32–35]. The slack particles also become active centers, and the activity of the deposition is increased. The treated alloy at 350°C has the best hydrogen evolution catalytic activity. It is believed that under this temperature condition, the structural relaxation of the amorphous structure completed, and the amorphous phase changes from one metastable state to another metastable structure. And the new metastable structure has higher activity energy. Thereby the treated alloy exhibited in higher catalytic activity and lower hydrogen evolution overpotential. The initial potential of the alloy electrode treated after 550°C is significantly reduced, and its catalytic activity is relatively weakened compared with the alloy containing the amorphous structure. It is considered that the existence of the amorphous structure increases the degree of disorder of the internal atomic arrangement. During the deposition progress, the H atom with larger activity and smaller radius is easily adsorbed in the disordered atomic cluster in the structure. Within the electrolysis process, it is easier to combine with itself to form an active center, and improve the catalytic activity of the alloy electrode. The increase of catalytic activity may also be a substitutional solid solution or intermetallic compound with more activity of [Mo] and [Ni] in the alloy coating with higher Mo content, and adsorbed H with smaller radius. The atom is solid-solubilized inside the alloy structure, and increases the electrocatalytic activity of the coating. The alloy electrode (Fig 5D) treated at 450°C in a short time has a higher catalytic activity and a lower hydrogen evolution overpotential at lower current density. It is considered that the amorphous crystallization process is unstable. The new phase crystal nucleus maintains the catalytic activity of the amorphous structure and exhibits a low hydrogen evolution overpotential. At a higher current density, the alloy quickly lost its own activity. The alloy electrode treated at 650°C (Fig 5F) has a sudden change in electrode performance at -1.45 V. This phenomenon suggests that the defects inside the crystalline structure still have higher energy and rapid migration ability during the electrolysis process, at lower current density, the defects in the crystal play a certain active center. When the current density is too large, the migration speed is lower than the hydrogen evolution rate, the voltage rises rapidly, and the energy consumption increases.

### 4.4 EIS properties analysis of alloy electrodes

**Fig 6:**
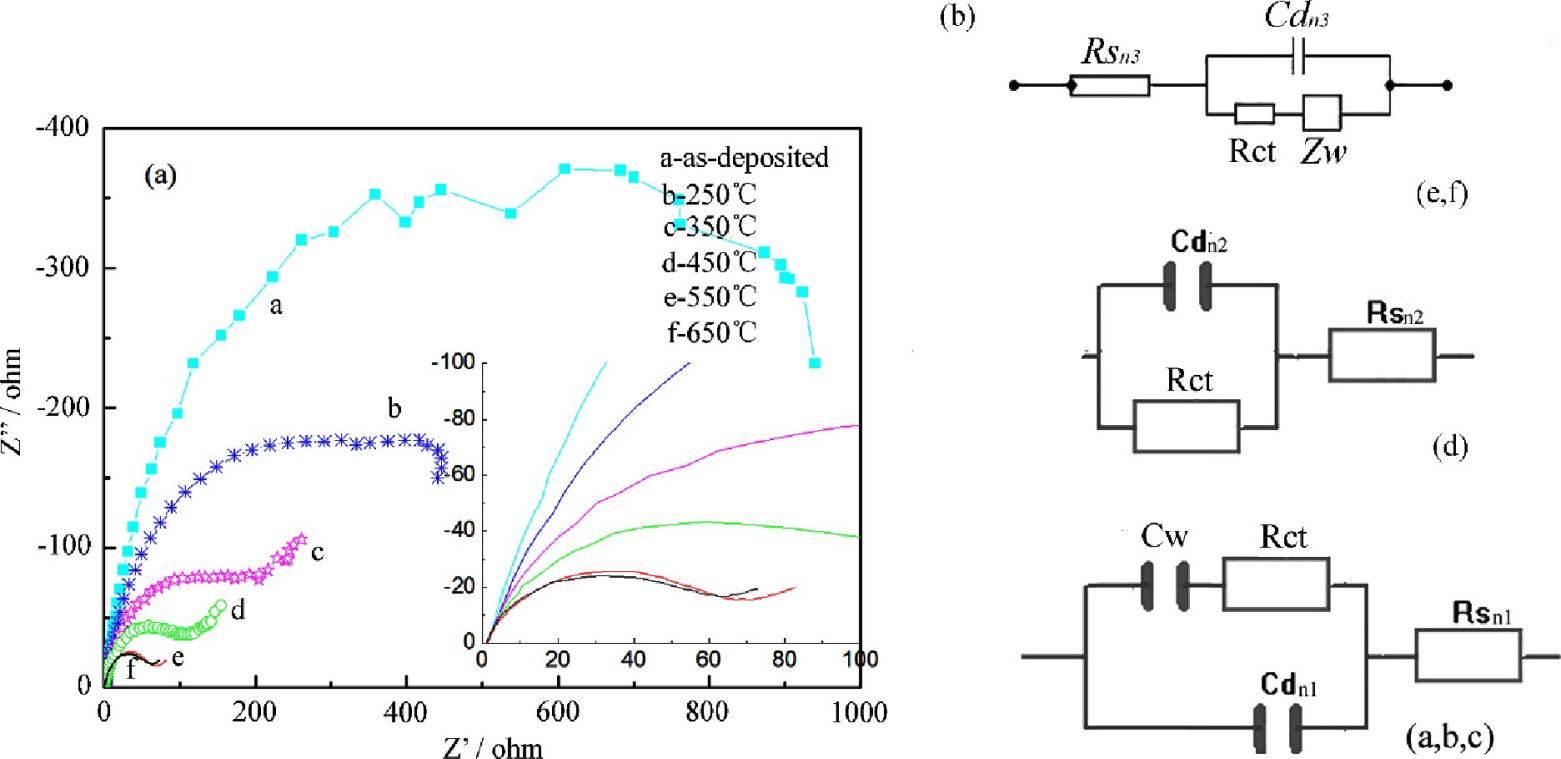
Electrochemical impedance spectroscopy for as-deposited and heat-treated samples (a) and equivalent circuit (b).

Fig 6 shows Electrochemical Impedance Spectroscopy (EIS) patterns of deposits as alloys electrodes at 33 ± 2°C, 7 mol/L NaOH solution. Fig 6(A) shows that the EIS spectrum of the crystalline alloy after heat treatment is a single semicircle. The hydrogen evolution process is a Volmer-Heyrovsky reaction to control the hydrogen evolution process, that is, the [H]atom in the adsorbed state is electrochemically desorbed to form hydrogen. The pattern of the mixed structure alloy is composed of a half circle and an approximate straight line, and the hydrogen evolution process could be a Volmer-Tafel mechanism, that is, the [H] atom in the adsorbed state is chemically desorbed to form H_2_ gas, and the rate determining step is a volmer reaction. The semicircle corresponds to the electrochemical reaction step of the electrode surface, and the semicircle diameter is approximately equal to the electrochemical reaction resistance Rr. The smaller the radius, the higher the electrochemical activity of the electrode specific surface. The analog equivalent circuit diagram for the Ni-Mo alloy is shown in Fig 6(B), and the equivalent parameters are shown in Table 3. Rs(n) represents the overall ohmic resistance of the electrolyte. Rct and Cd(n) are the charge transfer resistance and its related double-layer capacitance between the electrolyte and electrode. Zw is the Warburg impedance related to the diffusion of deposition in the electrode. Rct and Zw together constitute Faraday impedance, which reflects the dynamic reaction of the battery. And a high Rct generally corresponds to a relatively slow kinetics of the Faraday reaction. CPE is the smooth line or working electrode WE. Cw is the capacitance of the concentration polarization of the electrolyte. The difference between the Y-axis and X-axis value in the Nyquist curves may be related to material composition and coating structure. That is to say, the greater the difference between the component phases, the worse the stability of the coating structure and the matrix electrolyte, and the easier it is to cause the increase of polarization degree of the electrodes, which leads to the increase of mismatch degree between the electrodes. The materials microstructure XRD patterns after heat treatment show that when the temperature was higher than 450°C, the amorphous components in the film are almost absent. Fig 6(E) and 6(F) are in this state. No capacitive characteristics are shown in Electrochemical Impedance Spectroscopy of these two materials. Therefore, the fitting patterns are different with the incipient styles. And its CPE value cannot be obtained. From the fitting parameters in Table 3, the specific surface of the mixed-structure alloy increases with the increase of the amorphous content in the component, and the interaction contact between the electrode and the solution interface is increased after the heat treatment process. The resistance produces large impedance, while the generation of a new phase material with a finer grain size in the crystalline alloy increases the catalytic activity of the coating. Thereby, the increased contact surface accelerated the adsorbed [H] atom diffused, expedited hydrogen absorption and desorption, and increased the hydrogen evolution electrochemical activity of the electrode. The further growth of the crystal grains increases the crystal defects inside the deposits and increases the reactive center of the electrode after crystallization, and the new phase material changes the internal structure of the co-coating. As the degree of crystallization increases, the grain size becomes larger, the grain boundary length longer, the number of reactive centers decreases, and the catalytic activity decreases.

**Table 3:** Equivalent circuit parameters of the various components for as-deposited and heat-treated samples.

parameters electrodes	Rs /Ωcm^2^	Rct /Ωcm^2^	Cd (F/cm^2^)	CPE	S_real_ /cm^2^	amorphous content
650°C	5.12	60.60	3.306×10^−3^	-	165.3	-
550°C	5.54	67.42	3.564×10^−3^	-	178.2	-
450°C	6.43	167.87	3.786×10^−3^	-	189.3	37.82%
350°C	5.84	315.72	2.548×10^−3^	3.21×10^−3^	127.4	88.52%
As-deposited	7.46	1628.12	1.286×10^−3^	8.54×10^−3^	64.3	86.88%
250°C	9.46	667.87	1.134×10^−3^	7.23×10^−3^	56.7	87.34%

## 5. Conclusions

1. The nanocrystals in mixed structure coatings enlarges the relaxation temperature range of amorphous structure, prolongs the relaxation time, reduces the activation energy of crystallization of amorphous structure.
2. Mixed structural alloys have higher microhardness and better corrosion resistance than crystalline structural alloys, due to amorphous atomic clusters and a small amount of crystallographic defects in the alloy structure.
3. Under the condition of constant potential electrolysis, the mixed structure alloy has higher electrode catalytic activity and lower hydrogen evolution overpotential than the crystalline structure alloy, i.e. lower energy consumption, which is mainly caused by the active center of amorphous clusters and small size nanocrystals in the mixed structure.
4. The experimental data of AC impedance of alloy electrodes show that the mixed structure alloys have larger contact surface, lower self-resistance and higher electrocatalytic activity than crystalline alloys.
